# LETTER TO THE EDITOR The Perils of Do It Yourself Chemical Tattoo Removal

**Published:** 2010-03-12

**Authors:** Guang H. Yim, Sarah J. Hemington-Gorse, William A. Dickson

**Affiliations:** Welsh Centre for Burns and Plastic Surgery, Morriston Hospital, Swansea, SA6 6NL, United Kingdom

Dear Sir,

We would like to make members of the medical community aware of a problem that is increasing with the growth of the Internet. Regulating the Internet is a near impossibility and therefore patients are able to purchase a huge variety of products such as chemical peels that boast impressive results. Such Web sites are often accompanied by photographs of expected results, many of which are not truly representative of the product's action. With so many commodities available, it is not surprising that in the current climate of National Health Service (NHS) rationing of funding and treatments, patients turn to the Internet for further advice to self-manage their conditions.

We cite the case of a 26-year-old unemployed, heavily tattooed man who complained of psychological distress caused by tattoos on the dorsum of both hands. He claimed that these were the result of a practical joke while under the influence of alcohol. Although his GP was in the process of referring him for a plastic surgery consultation, he sought a more immediate solution by ordering a chemical peel from an unregulated Internet site that advertised the peel as “100% trichloroacetic acid (TCA) suitable for tattoo removal.” The Web site boasted excellent results following just one treatment.

Trichloroacetic acid is a chemical cauterant that coagulates the proteins of the skin and has been utilized for therapeutic treatment of dermatological conditions.^1^ It has also been proposed as a tattoo removal agent.^2^,^3^ However, as the concentration increases, the depth of dermal damage also increases.^1^ There are now many Web sites offering varying concentrations of TCA as a chemical peel or tattoo removal agent to the public as a nonprescribed product.^4^

The liquid arrived in an unmarked bottle with scant instructions for use and the patient applied the product for 10 minutes. After a few hours, he sought medical attention because the burning sensation would not subside. Following assessment, the patient was taken to the operation theatre for tangential excision and split skin grafting of both hands because of the deep nature of the burns (Figs 1 and 2). Preoperatively, we stressed that any treatment was for the chemical burn itself and not the removal of the tattoo.

Tattoos are a common adornment but are often regretted and therefore removal is a common request. Since this a nonessential service, the NHS Modernisation Agency has developed guidelines regarding access to this procedure.^5^ As a result, access to laser removal is restricted unless patients are prepared to fund the treatment privately. For many, this is not a financially viable option.

We feel that awareness needs to be raised for the problems associated with unlicensed product use and that Internet sites selling medical products need tighter regulation.

## Figures and Tables

**Figure 1:**
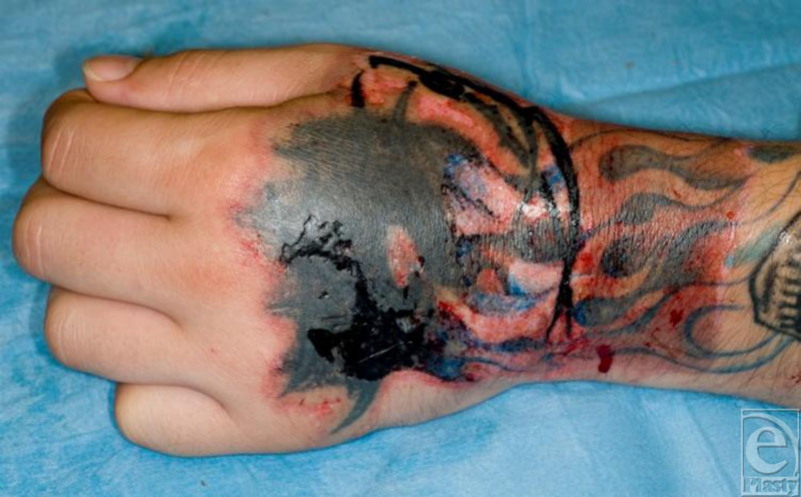
The chemical burn at 48 hours. Note that the presence of the tattoo made judging depth difficult.

**Figure 2:**
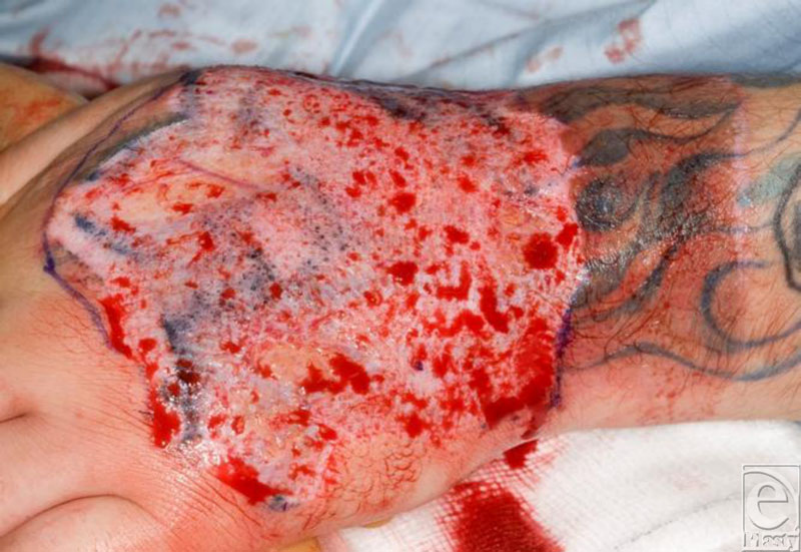
The burn depth following tangential excision is almost full thickness.

## References

[1] Collins P (1987). The chemical peel. Clin Dermatol.

[2] Hudson D, Lechtape-Gruter R (1990). A simple method of tattoo removal. S Afr Med J.

[3] Piggot T, Norris R (1988). The treatment of tattoos with trichloroacetic acid: experience with 670 patients. Br J Plast Surg.

[4] InkBusters.com, LLC Affordable tattoo removal. http://www.inkbusters.com.

[5] NHS Modernisation Agency Action on Plastic Surgery. Information for Commissioners of Plastic Surgery Services. Referrals and Guidelines in Plastic Surgery.

